# Benchmarking the Extent and Speed of Reperfusion: First Pass TICI 2c-3 Is a Preferred Endovascular Reperfusion Endpoint

**DOI:** 10.3389/fneur.2021.669934

**Published:** 2021-05-11

**Authors:** Albert J. Yoo, Jazba Soomro, Tommy Andersson, Jeffrey L. Saver, Marc Ribo, Hormozd Bozorgchami, Guilherme Dabus, David S. Liebeskind, Ashutosh Jadhav, Heinrich Mattle, Osama O. Zaidat

**Affiliations:** ^1^Department of Neurointervention, Texas Stroke Institute, Fort Worth, TX, United States; ^2^Neuroradiology, Karolinska University Hospital, Clinical Neuroscience Karolinska Institutet, Stockholm, Sweden; ^3^Medical Imaging, Allgemeine Ziekenhuis Groeninge, Kortrijk, Belgium; ^4^Department of Neurology, University of California, Los Angeles, Los Angeles, CA, United States; ^5^Stroke Unit, Department of Neurology, Vall D'Hebron University Hospital, Barcelona, Spain; ^6^Department of Neurology, Oregon Health and Science University Hospital, Portland, OR, United States; ^7^Department of Interventional Neuroradiology, Miami Cardiac and Vascular Institute at Baptist Hospital of Miami, Miami, FL, United States; ^8^Department of Neurology, Neurovascular Imaging Research Core and Stroke Center, University of California, Los Angeles, Los Angeles, CA, United States; ^9^Department of Neurology, University of Pittsburgh Medical Center, Pittsburgh, PA, United States; ^10^Department of Neurology, Inselspital, University of Bern, Bern, Switzerland; ^11^Mercy St. Vincent Medical Center, Toledo, OH, United States

**Keywords:** intra-arterial therapy, reperfusion grading, reperfusion, brain ischaemia, cerebral infacrction, stent retriever, mechanical thrombectomy

## Abstract

**Background and Purpose:** End-of-procedure substantial reperfusion [modified Treatment in Cerebral Ischemia (mTICI) 2b-3], the leading endpoint for thrombectomy studies, has several limitations including a ceiling effect, with recent achieved rates of ~90%. We aimed to identify a more optimal definition of angiographic success along two dimensions: (1) the extent of tissue reperfusion, and (2) the speed of revascularization.

**Methods:** Core-lab adjudicated TICI scores for the first three passes of EmboTrap and the final all-procedures result were analyzed in the ARISE II multicenter study. The clinical impact of extent of reperfusion and speed of reperfusion (first-pass vs. later-pass) were evaluated. Clinical outcomes included 90-day functional independence [modified Rankin Scale (mRS) 0–2], 90-day freedom-from-disability (mRS 0–1), and dramatic early improvement [24-h National Institutes of Health Stroke Scale (NIHSS) improvement ≥ 8 points].

**Results:** Among 161 ARISE II subjects with ICA or MCA M1 occlusions, reperfusion results at procedure end showed substantial reperfusion in 149 (92.5%), excellent reperfusion in 121 (75.2%), and complete reperfusion in 79 (49.1%). Reperfusion rates on first pass were substantial in 81 (50.3%), excellent reperfusion in 62 (38.5%), and complete reperfusion in 44 (27.3%). First-pass excellent reperfusion (first-pass TICI 2c-3) had the greatest nominal predictive value for 90-day mRS 0–2 (sensitivity 58.5%, specificity 68.6%). There was a progressive worsening of outcomes with each additional pass required to achieve TICI 2c-3.

**Conclusions:** First-pass excellent reperfusion (TICI 2c-3), reflecting rapid achievement of extensive reperfusion, is the technical revascularization endpoint that best predicted functional independence in this international multicenter trial and is an attractive candidate for a lead angiographic endpoint for future trials.

**Clinical Trial Registration:** http://www.clinicaltrials.gov, identifier NCT02488915.

## Introduction

The current consensus statement-endorsed benchmark for procedural success after intra-arterial stroke therapy (IAT) is procedure end substantial reperfusion [modified Treatment in Cerebral Ischemia (mTICI) score of 2b or higher], defined as the restoration of anterograde tissue perfusion in more than 50% of the target downstream territory (1, 2). The impressive clinical benefits observed in recent thrombectomy trials reflected improved reperfusion with second generation devices, most notably stent retrievers (3, 4). Since these pivotal trials, there has been a further increase in reported rates of substantial reperfusion (5). In the recent ARISE (Analysis of Revascularization in Ischemic Stroke with EmboTrap) II study, the core lab-adjudicated TICI 2b-3 rate at procedure end was 92.5% (6).

However, there are considerable limitations to using the rate of TICI 2b-3 as a lead technical efficacy endpoint for IAT trials. First, this endpoint counts moderate reperfusion as a success, but when reperfusion is only 50–90% achieved, substantial tissue volumes remain in jeopardy. Second, the outcome of TICI 2b-3 is considered a success regardless of the number of passes required to achieve it, but maximal benefit is likely to be conferred by first-pass success, thus reducing ischemia duration (7). Third, the high rate of TICI 2b-3 seen with modern endovascular technology results in a ceiling effect, making the measure insensitive to further improvements in endovascular technique. Accordingly, a reevaluation of the optimal angiographic endpoint is necessary.

Using core-lab adjudicated data from ARISE II, we aimed to assess the clinical impact of the first-pass effect (FPE) and to identify the optimal definition of angiographic success along two dimensions: (1) the extent of tissue reperfusion, and (2) the speed of revascularization.

## Materials and Methods

All data generated or analyzed during this study are included in this published article and its Supplementary Material. The ARISE II study design and methods have been previously described (6). The study protocol was approved by the institutional review board/ethics committee at each participating site. All patients or their legally authorized representatives provided written informed consent before enrollment. To analyze a cohort with similar relationships between perfusion deficits and outcomes, only anterior circulation occlusions were included. To limit variability in the size of the at-risk territory, M2 occlusions were excluded, leaving only internal carotid artery (ICA) and middle cerebral artery (MCA) M1 occlusions in the study cohort. Angiographic endpoints were core lab-adjudicated and included TICI scores after each of the first three EmboTrap passes and final TICI score after all interventions. TICI scoring was inclusive of the 2c score (near complete or >90% reperfusion of the downstream territory). Core lab readers were blinded to clinical outcomes.

### Statistical Analysis

Baseline characteristics were reported using standard descriptive statistics. Parametric and non-parametric methods were applied where appropriate.

The clinical impact of first-pass reperfusion was measured controlling for final reperfusion grade. For example, among subjects with final TICI score of 3, the subgroup where TICI 3 was achieved on the first pass was compared with the subgroup requiring multiple passes. These subgroups were compared on four efficacy and two safety outcomes. The primary efficacy outcome was 90-day functional independence [modified Rankin Scale (mRS) 0–2]. Additional efficacy outcomes were 90-day freedom from disability (mRS 0–1), 90-day level of disability (ordinal 6-level mRS), and dramatic early improvement [24-h National Institutes of Health Stroke Scale (NIHSS) improvement ≥8 points]. Safety outcomes were symptomatic intracranial hemorrhage (sICH) and 90-day mortality. Endpoints were assessed using the Chi-squared, Fisher's Exact, or Wilcoxon rank sum test as appropriate. This analysis was similarly done for subjects with final TICI 2c and separately 2b. Multiple logistic regression analysis for 90-day mRS 0–2 was performed to assess the impact of first-pass success adjusting for covariates with univariate *P* < 0.1.

To explore the effect of final reperfusion extent on functional outcome, 90-day mRS scores were compared between final TICI grades using the Jonckheere-Terpstra trend test. Receiver-operating characteristic (ROC) analysis was used to identify the optimal TICI threshold for discriminating 90-day mRS 0–2 and 0–1, and 24-h NIHSS improvement of 8 or more points.

The extent of reperfusion at procedure end that was most strongly associated with outcomes was assessed by comparing: substantial reperfusion (mTICI 2b-3), excellent reperfusion (TICI 2c-3), and complete reperfusion (TICI 3). The extent measure that nominally performed best was then further tested to assess the impact of speed of attainment, by comparing achievement on first, second, third, or fourth or higher passes.

Because the core lab assessed reperfusion after each of the first three passes and at procedure end, all passes beyond the third were aggregated into a single category. The effect of the number of passes on 90-day mRS was evaluated using the Jonckheere-Terpstra trend test. ROC analysis was used to identify the optimal number of passes for predicting 90-day functional independence, 90-day freedom-from-disability and 24-h dramatic neurologic improvement. In all ROC analyses, the optimal operating point was defined as the point with the maximum Youden index (=sensitivity + specificity − 1). Statistical significance was defined as two-tailed *P*-value <0.05.

Statistical analysis was performed using MedCalc Software version 19 (Ostend, Belgium). The conclusions were verified by an independent statistician using SAS version 9.4 software (Cary, NC).

ARISE II was sponsored by Neuravi, Inc., currently Cerenovus/Johnson & Johnson. This study is the academic work of the authors. The sponsor played no role in the design and conduct of the study; collection, management, analysis, and interpretation of the data; preparation, review, or approval of the manuscript; and decision to submit the manuscript for publication.

## Results

### Overall Angiographic Results

Two hundred twenty-seven patients were treated with EmboTrap in ARISE II. Nine basilar occlusion patients and 57 M2 occlusion patients were removed for this analysis. Baseline characteristics and outcomes are provided in Table 1. Of the 161 patients meeting study entry criteria, 149 (92.5%) patients had substantial reperfusion at procedure end (final TICI 2b-3): 28 (17.4%) final TICI 2b; 42 (26.1%) final TICI 2c; and 79 (49.1%) final TICI 3. The median number of thrombectomy passes was 2 [interquartile range (IQR) 1–3], and the highest number of passes was 9. Median procedural time was 44 (IQR 27–70) min.

**Table 1:** Baseline characteristics of study population (*n* = 161).

**Variable**	
Age (years); mean ± SD	66.7 ± 13.3
Female sex; *n* (%)	92 (57.1%)
Baseline NIHSS score; median (IQR)	16 (13–20)
Baseline NCCT ASPECTS; median (IQR) (*n* = 124)	10 (10–10)
**Occlusion level;** ***n*** **(%)**	
ICA	35 (21.7%)
MCA M1	126 (78.3%)
IV tPA treatment; *n* (%)	106 (65.8%)
Hypertension; *n* (%)	107 (66.5%)
Diabetes mellitus; *n* (%)	31 (19.3%)
Atrial fibrillation; *n* (%)	62 (38.5%)
Dyslipidemia; *n* (%)	63 (39.1%)
Smoking; *n* (%)	42 (26.1%)
Previous stroke/transient ischemic attack; *n* (%)	29 (18.0%)
Previous MI/CAD; *n* (%)	35 (21.7%)
**Clinical and safety outcomes**	
24-h NIHSS score; median (IQR) (*n* = 155)	4 (1–14)
Dramatic neurologic improvement [Baseline to 24-h NIHSS score improvement ≥ 8 points; *n* (%)]	94/155 (60.6%)
90-day disability level mRS; median (IQR) (*n* = 156)	1.5 (0–4)
90-day functional independence, mRS 0–2; *n* (%)	103/156 (66.0%)
90-day freedom-from-disability, mRS 0–1; *n* (%)	78/156 (50.0%)
90-day mortality; *n* (%)	14/156 (9.0%)
sICH; *n* (%)	10 (6.2%)

*SD, standard deviation; NIHSS, National Institutes of Health Stroke Scale; IV tPA, intravenous tissue plasminogen activator; IQR, interquartile range; MI/CAD, myocardial infarction/coronary artery disease; mRS, modified Rankin Scale; NCCT ASPECTS, non-contrast CT Alberta Stroke Program Early CT Score; sICH, symptomatic intracranial hemorrhage*.

Substantial reperfusion (TICI 2b-3) after the first pass was seen in 81 (50.3%) patients: 19 (11.8%) first-pass TICI 2b; 18 (11.2%) first-pass TICI 2c; and 44 (27.3%) first-pass TICI 3. In the remainder, there were 53 (32.9%) first-pass TICI 0–1 and 27 (16.8%) first-pass TICI 2a. Median procedural time from groin puncture to achieving TICI ≥ 2b was 27 (IQR 22–38) min in the subjects who had first-pass TICI 2b-3 vs. 61 (IQR 46–86.5) min in those who achieved TICI 2b-3 after two or more passes (*P* < 0.0001).

### First-Pass Success vs. Multiple Passes: Final Angiographic and Clinical Outcomes

**Figure 1:**
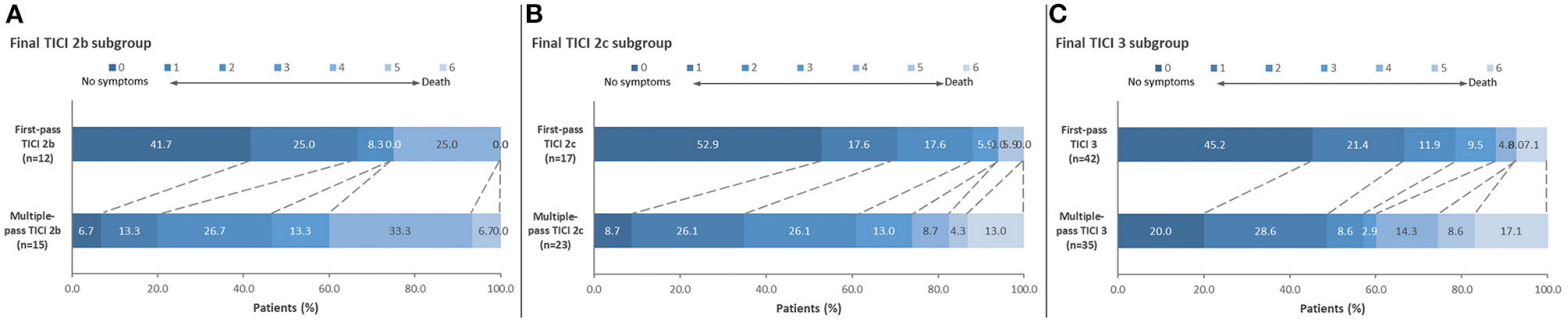
Distribution of 90-day mRS scores comparing first-pass reperfusion vs. multiple-pass reperfusion. Results are shown for **(A)** final TICI 2b: median 90-day mRS scores were 1 (IQR 0-3) vs. 3 (IQR 2–4) for the first-pass group vs. the multiple-pass group (*P* = 0.04); **(B)** final TICI 2c: median 90-day mRS scores were 0 (IQR 0–2) vs. 2 (IQR 1–4) for the first-pass group vs. the multiple-pass group (*P* = 0.004); and **(C)** final TICI 3 patients: median 90-day mRS scores were 1 (IQR 0–2) vs. 2 (IQR 1–5) for the first-pass group vs. the multiple-pass group (*P* = 0.01).

Supplementary Material enumerates the breakdown of final TICI results based on the first pass TICI score. When controlling for final reperfusion grade, there were significantly better 90-day ordinal mRS outcomes among patients who achieved their final TICI score on the first pass compared to multiple passes (Figure 1). For final TICI 2b, the median 90-day mRS scores were 1 (IQR 0–3) vs. 3 (IQR 2–4) for the first-pass group vs. the multiple-pass group (*P* = 0.04). For final TICI 2c, the median 90-day mRS scores were 0 (IQR 0–2) vs. 2 (IQR 1–4) (first-pass group vs. the multiple-pass group; *P* = 0.004). For final TICI 3, the median 90-day mRS scores were 1 (IQR 0–2) vs. 2 (IQR 1–5) (first-pass group vs. the multiple-pass group; *P* = 0.01). There were no significant differences among the first-pass and multiple-pass groups for sICH and for 90-day mortality, although there were numerically more safety events in the multiple-pass groups in most cases (Table 2). After adjusting for age, baseline NIHSS score, baseline Alberta Stroke Program Early Computed Tomography Score (ASPECTS), vessel occlusion level, atrial fibrillation, and final TICI 2c-3 score, first-pass success (i.e., when the final TICI score is achieved on the first pass) was an independent predictor of 90-day mRS 0–2 [odds ratio (OR) 3.42 (95% CI, 1.27–9.17), *P* = 0.01]. The associations between the baseline variables and both 90-day mRS 0–2 and final TICI 2c-3 are provided in Supplementary Material.

**Table 2:** Dichotomized clinical and safety endpoints for first-pass vs. multiple-pass final TICI scores.

	**Final 2b, FP 2b**	**Final 2b, not FP 2b**	***P*-value**	**Final 2c, FP 2c**	**Final 2c, not FP 2c**	***P*-value**	**Final 3, FP 3**	**Final 3, not FP 3**	***P*-value**
90-day mRS0–2	9/12 (75%)	7/15 (46.7%)	0.24	15/17 (88.2%)	14/23 (60.9%)	0.08	33/42 (78.6%)	20/35 (57.1%)	0.04
90-day mRS0–1	8/12 (66.7%)	3/15 (20%)	0.02	12/17 (70.6%)	8/23 (34.8%)	0.03	28/42 (66.7%)	17/35 (48.6%)	0.11
24-hr NIHSS drop 8+ pts	8/13 (61.5%)	6/15 (40%)	0.26	14/18 (77.8%)	12/24 (50%)	0.07	29/40 (72.5%)	21/34 (61.8%)	0.33
sICH	0/13	2/15 (13.3%)	0.48	0/18	3/24 (12.5%)	0.25	1/44 (2.3%)	1/35 (2.9%)	1.00
90-daymortality	0/12	0/15	NC	0/17	3/23 (13.0%)	0.25	3/42 (7.1%)	6/35 (17.1%)	0.17

*FP, first-pass; mRS, modified Rankin Scale; NIHSS, National Institutes of Health Stroke Scale; NC, not calculable; TICI, Treatment in Cerebral Ischemia scale*.

### Optimal Final TICI Score for Discriminating Good Clinical Outcome

**Figure 2:**
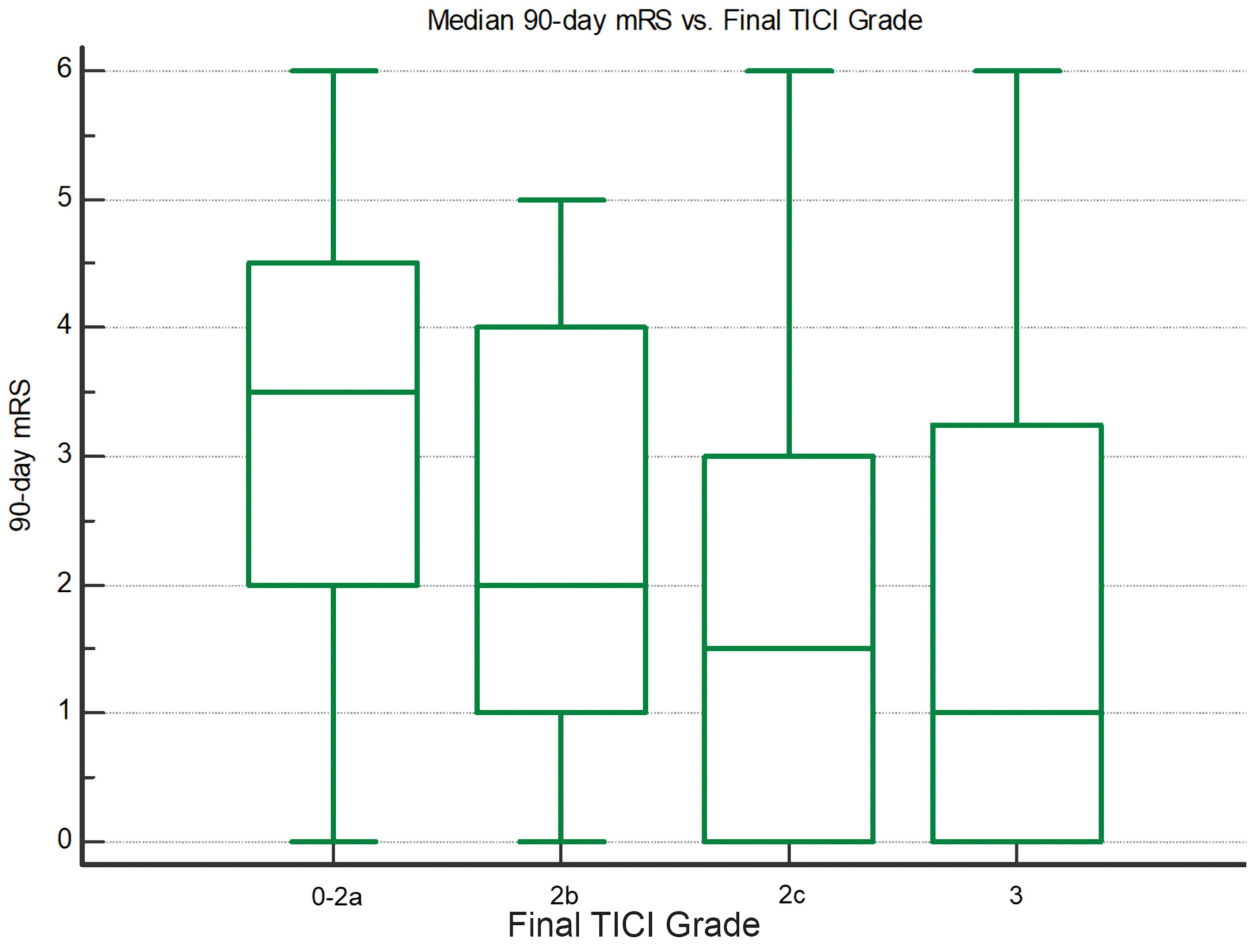
Box-whisker plots showing median 90-day mRS vs. final TICI grade. There are significantly lower mRS scores with greater final reperfusion (*P* < 0.05; Jonckheere-Terpstra trend test).

90-day functional outcome was significantly better with greater final reperfusion extent (Figure 2). Median 90-day mRS was 1 (IQR 0–3.25) for final TICI 3, 1.5 (IQR 0–3) for TICI 2c, 2 (IQR 1–4) for TICI 2b, and 3.5 (IQR 2–4.5) for TICI 0-2a (*P* < 0.05; Jonckheere-Terpstra trend test). Final TICI 2c-3 showed the highest nominal accuracy for discriminating 90-day mRS 0–2 (sensitivity 79.6%, specificity 34.0%) and mRS 0–1 (sensitivity 83.3%, specificity 33.3%), and 24-h NIHSS improvement ≥8 points (sensitivity 80.9%, specificity 34.4%).

### Optimal Number of Passes for Achieving Final TICI 2c-3

**Figure 3:**
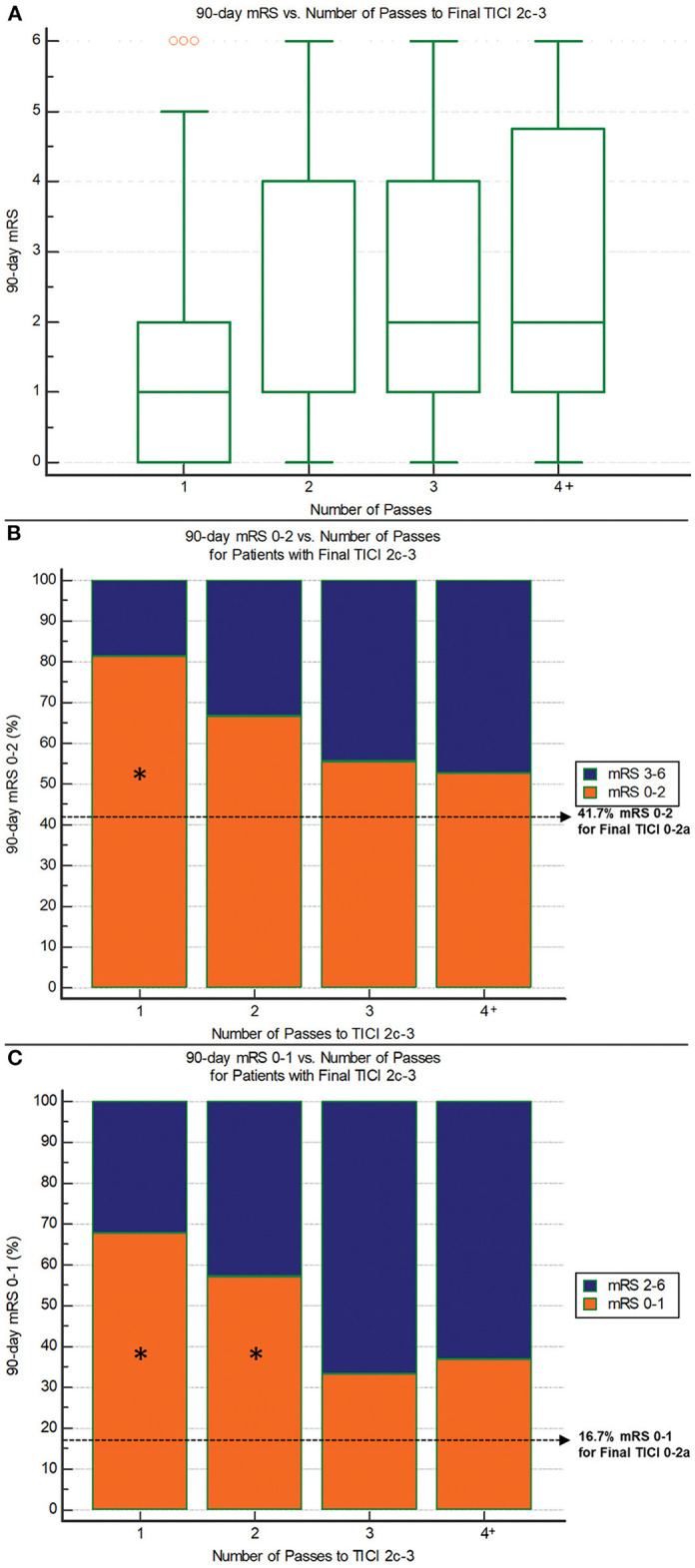
90-day outcomes vs. the number of passes required to reach final TICI 2c-3 reperfusion. **(A)** 90-day mRS: There are significantly lower mRS scores with fewer passes (*P* = 0.0001; Jonckheere-Terpstra trend test). **(B)** 90-day mRS 0–2: There are progressively lower rates of good outcome with increasing number of passes (*P* = 0.005; Chi-squared test for trend). Asterisk indicates significant difference compared to non-reperfusers (final TICI 0-2a). **(C)** 90-day mRS 0–1: There are progressively lower rates of excellent outcome with increasing number of passes (*P* = 0.003; Chi-squared test for trend). Asterisk indicates significant difference compared to non-reperfusers (final TICI 0-2a).

The median number of passes for patients with final TICI 2c-3 was one (IQR 1–3). Figure 3A illustrates the relationship between number of passes for achieving TICI 2c-3 and 90-day functional outcome. Median 90-day mRS was 1 (IQR 0–2) for one pass, 1 (IQR 1–4) for two passes, 2 (IQR 1–4) for three passes, and 2 (IQR 1–5) for four or more passes (*P* = 0.0001; Jonckheere-Terpstra trend test). Median procedure time for one pass was 25 (IQR 20–34) min, for two passes was 44 (IQR 35–57.5) min, for three passes was 60 (IQR 48–64) min, and for four or more passes was 76 (IQR 64.5–105) min (*P* < 0.00001; Jonckheere-Terpstra trend test). One pass showed the highest nominal accuracy for discriminating 90-day mRS 0–2 (sensitivity 58.5%, specificity 68.6%) and 24-h NIHSS score improvement ≥8 points (sensitivity 56.6%, specificity 62.5%). Importantly, there were no significant imbalances in the baseline variables between first-pass TICI 2c-3 and non-first-pass TICI 2c-3 (Table 3). Two or fewer passes was the optimal threshold for discriminating 90-day mRS 0–1 (sensitivity 80.0%, specificity 46.2%).

**Table 3:** Comparison of baseline variables between first-pass TICI 2c-3 vs. non-first-pass TICI 2c-3.

**Variable**	**FP TICI 2c-3 (*n* = 62)**	**Non-FP TICI 2c-3 (*n* = 59)**	***P*-value**
Age (years); mean ± SD	67.2 ± 12.8	66.6 ± 14.0	0.78
Female sex; *n* (%)	36 (58.1%)	36 (61.0%)	0.74
Baseline NIHSS score; median (IQR)	16.5 (12–19)	17 (14–21)	0.36
Baseline NCCT ASPECTS; median (IQR) (*n* = 94)	10 (9.5–10) (*n* = 48)	10 (9–10) (*n* = 46)	0.80
**Occlusion level;** ***n*** **(%)**			
ICA	14 (22.6%)	13 (22.0%)	0.94
MCA M1	48 (77.4%)	46 (78.0%)	
IV tPA treatment; *n* (%)	38 (61.3%)	44 (74.6%)	0.12
Hypertension; *n* (%)	44 (71.0%)	40 (67.8%)	0.71
Diabetes mellitus; *n* (%)	12 (19.4%)	11 (18.6%)	0.92
Atrial fibrillation; *n* (%)	22 (35.5%)	25 (42.4%)	0.44
Dyslipidemia; *n* (%)	22 (35.5%)	26 (44.1%)	0.34
Smoking; *n* (%)	13 (21.0%)	18 (30.5%)	0.23
Previous stroke/transient ischemic attack; *n* (%)	11 (17.7%)	9 (15.3%)	0.71
Previous MI/CAD; *n* (%)	18 (29.0%)	11 (18.6%)	0.18

*FP, first-pass; IV tPA, intravenous tissue plasminogen activator; IQR, interquartile range; MI/CAD, myocardial infarction/coronary artery disease; NCCT ASPECTS, non-contrast CT Alberta Stroke Program Early CT Score; NIHSS, National Institutes of Health Stroke Scale; SD, standard deviation; TICI, Treatment in Cerebral Ischemia scale*.

Regarding safety, each subsequent pass required to achieve TICI 2c-3 was associated with higher mortality: 90-day mortality was 5.1% (3/59) for one pass, 9.5% (2/21) for two passes, 16.7% (3/18) for three passes, and 21.1% (4/19) for four or more passes (*P* = 0.03, Chi-squared trend test). For comparison, mortality in those without reperfusion (TICI 0-2a) was 16.7%. There was no significant relationship between number of passes and sICH. Rates of sICH were 1.6% (1/62) for one pass, 4.8% (1/21) for two passes, 10.5% (2/19) for three passes, and 5.3% (1/19) for four or more passes (*P* = 0.20, Chi-squared trend test). The sICH rate for TICI 0-2a patients was 25% (3/12).

The impact of the number of passes to final TICI 2c-3 on 90-day outcome is shown separately for patients treated early from stroke onset (≤4 h to groin puncture) vs. late (>4 h) in Supplementary Material.

### Likelihood of Achieving Final TICI 2c-3 Based on First-Pass Result

There was a significantly higher chance of achieving TICI 2c-3 on the first pass [38.5% (62/161)] compared to after the first pass [27.1% (59/217); *P* = 0.02]. Furthermore, if the first pass did not result in TICI 2c-3 and further attempts were performed, there was a higher chance of achieving final TICI 2c-3 when the first pass yielded a lower reperfusion grade. Rates of final TICI 2c-3 were 71.7% (38/53) for first-pass TICI 0-1, 55.6% (15/27) for first-pass TICI 2a, and 50% (6/12) for first-pass TICI 2b (*P* = 0.08; Chi-squared test for trend). There were more overall passes for lower first-pass TICI scores: median 3 (IQR 3–5) for first-pass TICI 0–1 vs. 3 (IQR 2–3) for first-pass TICI 2a vs. 2 (IQR 2–2.5) for first-pass TICI 2b (*P* = 0.0004; Jonckheere-Terpstra trend test).

## Discussion

Core-lab adjudicated ARISE II data confirm the superior clinical benefit of first-pass reperfusion. When adjusting for final TICI score, first-pass success (defined as when the final TICI score of 2b-3 is achieved on the first pass) yielded significantly better 90-day functional outcomes and was an independent predictor of 90-day independence. Furthermore, first-pass TICI 2c-3 was the optimal combination of reperfusion extent and speed for predicting good outcome after IAT. These findings were similar when restricting the analysis to subjects who received 3 or fewer passes with EmboTrap.

The FPE has been variably defined as first-pass TICI 3 and more recently first-pass TICI 2c-3 (8–10). Our study supports the inclusion of 2c scores [defined in ARISE II as >90% anterograde reperfusion (1)] into a standardized FPE definition. Final TICI 2c-3 was achieved in 75.2% of the study cohort compared to 49.1% for TICI 3 alone and provided the best discrimination of good and excellent 90-day functional outcomes (mRS 0–1 and 0–2) and early dramatic neurologic improvement (24-h NIHSS improvement ≥8 points). This mirrors previous reports that have found similar outcomes between TICI 2c and 3 reperfusion (11, 12). In an ancillary analysis of the ASTER trial, the magnitude of benefit for achieving 90-day mRS 0–2 was congruent between TICI 2c and 3 relative to TICI 2b, and combined TICI 2c-3 patients had a significantly higher rate of favorable outcomes compared to 2b patients [OR 1.72 (95% CI, 1.01–2.90)] (12).

Another endpoint used in recent studies is first-pass TICI 2b-3, termed modified FPE (8, 9). A major limitation of this endpoint is that TICI 2b encompasses an overly broad range of reperfusion results (50–89% of the ischemic territory), many of which might be considered suboptimal currently. It is likely for this reason that the majority of first-pass TICI 2b patients [12/19 (63%)] underwent additional passes, questioning the clinical relevance of this first-pass category. There may be potential value of the expanded TICI (eTICI) scale, which subdivides TICI 2b grades into eTICI 2b50 (50–66% reperfusion) and 2b67 (67–89% reperfusion). In the HERMES dataset, the c-statistic for discriminating 90-day mRS 0–2 was slightly higher for eTICI (0.664) compared to the TICI classification employed in ARISE II (0.661), the only difference being the 2b67 categorization (13). Future studies should investigate whether a reformulation of FPE to include eTICI 2b67 results is warranted.

An additional argument against including TICI 2b scores into the FPE definition is the issue of clot fragmentation, which can impede full reperfusion. Our analysis revealed a numerically lower likelihood of achieving TICI 2c-3 when the first pass yielded TICI 2b compared to a lesser score. Among patients who underwent multiple passes, there was a stepwise reduction in final TICI 2c-3 rate from a first-pass TICI score of 0–1 (72%) to 2a (56%) to 2b (50%) (*P* = 0.08). This observation likely owes to the smaller, more distal vessel segments that remain occluded when there is greater partial reperfusion, which are more difficult and riskier to treat. This increased risk may explain why there were fewer passes performed after first-pass TICI 2a and fewer still after TICI 2b compared to TICI 0–1. In this context, both TICI 2a and 2b may be viewed as unwelcome indicators of thrombus fragmentation. Thrombus friability likely plays a central role in cases of distal embolization, and techniques should be targeted to minimize this phenomenon.

The optimal speed for attaining TICI 2c-3 was one thrombectomy pass (first-pass TICI 2c-3), which was observed in 51.2% of final TICI 2c-3 patients and best predicted 90-day good outcome and early dramatic neurological improvement compared to other numbers of passes. There was a worsening of clinical outcomes with each additional pass, consistent with prior work showing that procedural time to reperfusion is a powerful predictor of IAT outcomes (7). With regards to benchmarking device performance, the number of passes is a more suitable measure of device-related revascularization speed than procedure time because it disregards the time required for vessel catheterization, a delay which is unrelated to device action and can be highly variable (14).

A substantial proportion of study patients required three or more thrombectomy passes (37% in the entire cohort, and 31% among those achieving final TICI 2c-3). In refractory cases, an important question facing neurointerventionists is how many passes should be performed before stopping. A recent study of stent retriever thrombectomy reported dismal outcomes after the third pass (7.4% rate of 90-day mRS 0–2 for passes 4 through 8) despite an ~25% per-pass rate of TICI 2b-3 (15). Conversely, another study of largely stent retrievers (95%) found a significantly higher rate of good outcome in patients who achieved TICI 2b-3 on the fourth pass compared to non-reperfusers (16). Our analysis also suggests clinical value in pursuing more than three passes. First, even though the chance of achieving TICI 2c-3 dropped after the first pass, there was still a 30% per-pass rate of TICI 2c-3 among passes 3 or greater. And when TICI 2c-3 is achieved, there are higher rates of 90-day mRS 0–2 and 0–1 (Figures 3A–C) for every pass number, including four or higher, compared to non-reperfusers (final TICI 0-2a). Although many of these per-pass comparisons were not statistically significant, this may be due to small sample sizes. The clinical value of near-complete/complete reperfusion achieved in a delayed fashion is consistent with other studies showing that complete reperfusion mitigates the deleterious effect of treatment delay (16, 17). In addition, there were no obvious safety concerns with pursuing more than three passes. Although mortality increased with each additional pass required to achieve TICI 2c-3, the mortality associated with 4 or more passes (21.1%) was comparable to that seen in non-reperfusers (16.7%). Concerning sICH, there was no significant association with pass number, and the rate of sICH with 4 or more passes (5.3%) was lower than that seen in non-reperfusers (25%). Previous reports are contradictory regarding the relationship between the number of passes and hemorrhagic conversion (18, 19).

Study limitations include use of a single device for the first three thrombectomy passes, which may limit generalizability. However, it is likely that the principal findings regarding the relationship between clinical outcome and the extent and speed of reperfusion are independent of how this reperfusion is achieved. Another limitation is that the study cohort comprised largely ideal treatment candidates, as reflected in their baseline ASPECTS scores (76% with ASPECTS 10). As such, the outstanding outcomes reported here (66% mRS 0–2 and 50% mRS 0–1 at 90 days) do not reflect real-world practice. These limitations stem from the ARISE II study design as a prospective registration trial for FDA approval of EmboTrap. However, this design also lent numerous strengths to the analysis, including rigorous data collection and monitoring, minimal subject attrition (3% at 90 days), and strict core lab adjudication of reperfusion results. Unlike previous thrombectomy studies, the ARISE II core lab prospectively evaluated each of the first three thrombectomy passes, yielding novel core lab-adjudicated data concerning reperfusion speed.

## Conclusions

Data from ARISE II underscore the critical impact of procedural time to reperfusion on clinical outcomes after thrombectomy. First-pass TICI 2c-3 provides the optimal measure of both extent and speed of reperfusion for predicting good functional outcome and may serve as a useful benchmark for testing device performance and thrombectomy techniques in future studies.

## Data Availability Statement

The original contributions presented in the study are included in the article/Supplementary Material, further inquiries can be directed to the corresponding author/s.

## Ethics Statement

The studies involving human participants were reviewed and approved by the institutional review board/ethics committee at each participating site. The patients/participants provided their written informed consent to participate in this study.

## Author Contributions

AY contributed to the conceptualization, methodology, software, formal analysis, and writing of the original draft of the manuscript. AY, TA, JSa, MR, HB, GD, DL, AJ, HM, OZ, and ARISE II Investigators participated in the investigation and provided resources and project administration. JSo contributed to the data visualization. AY, TA, JSa, MR, HB, HM, and OZ provided study supervision. AY, JSo, TA, JSa, MR, HB, GD, DL, AJ, HM, and OZ contributed to the review and editing of the final draft of the manuscript. All authors contributed to the article and approved the submitted version.

## Conflict of Interest

AY is a consultant for Cerenovus, Penumbra, and Zoll, receives research grants from Medtronic, Cerenovus, Penumbra, Stryker, and Genentech, and has equity interest in Insera Therapeutics. TA is a consultant for Neuravi, Ablynx, Amnis Therapeutics, Medtronic, Rapid Medical, and Stryker. The University of California, Regents receives funding for JSa's services as a scientific consultant regarding trial design and conduct for Covidien and Stryker; JSa is an employee of the University of California, which holds a patent on retriever devices for stroke. MR is a shareholder in Anaconda Biomed, consultant for Cerenovus, Medtronic, Stryker, Apta Targets, and Vesalio. HB serves as a modest consultant for Cerenovus/Neuravi, and Stryker. GD serves as a consultant for Medtronic, Microvention, Penumbra, and Cerenovus. DL serves as an imaging core lab consultant for Cerenovus, Genentech, Medtronic, Stryker, and Vesalio. HM reports personal fees from Covidien/Medtronic, Neuravi/Cerenovus, Servier, and Bayer outside the submitted work, and served on the steering committees of the SWIFT PRIME and ARISE studies. OZ serves as a consultant for Neuravi, Stryker, Penumbra, and Medtronic. The remaining authors declare that the research was conducted in the absence of any commercial or financial relationships that could be construed as a potential conflict of interest.

## Abbreviations

ARISE II: Analysis of Revascularization in Ischemic Stroke with EmboTrap II study
ASPECTS: Alberta Stroke Program Early CT Score
ASTER: Direct Aspiration First Pass Technique for Thrombectomy Revascularisation of Large Vessel Occlusion in Acute Ischaemic Stroke
FP: first pass
FPE: first pass effect
HERMES: Highly Effective Reperfusion evaluated in Multiple Endovascular Stroke Trials Collaboration
IAT: intra-arterial stroke therapy
ICA: internal carotid artery
IQR: interquartile range
MCA: middle cerebral artery
mTICI: modified Treatment in Cerebral Ischemia
ROC: receiver-operating characteristic curve
sICH: symptomatic intracranial hemorrhage
TICI: Treatment in Cerebral Ischemia.
