# Supplementary material for: Benchmarking the Extent and Speed of Reperfusion: First Pass TICI 2c-3 Is a Preferred Endovascular Reperfusion Endpoint
Source: Front Neurol. 2021 May 11;12:669934. doi: 10.3389/fneur.2021.669934 (PMC8144635; doi:10.3389/fneur.2021.669934)
Supplement: Supplementary file 1 [file Data_Sheet_1.doc]

Supplementary Material

**Contents**

[Supplemental Table I. First pass vs. Final TICI results 2](#__RefHeading___Toc64621331)

[Supplemental Table II. Association of baseline variables with clinical and final angiographic outcomes 3](#__RefHeading___Toc64621332)

[Supplemental Table III. Number of passes to final TICI 2c-3 vs. 90-day functional outcome in early- vs. late-treated patients. 4](#__RefHeading___Toc64621333)

[Supplemental Figure I: Study Flowchart 5](#__RefHeading___Toc64621334)

Supplemental Figure II: ROC Curve for Final TICI (0-2a vs. 2b vs. 2c vs. 3) versus 90-day mRS 0-2………..6

Supplemental Figure III: ROC Curve for Number of Passes to TICI 2c-3 (1 vs. ≤2 vs. ≤3 vs. 4+) versus 90-day mRS 0-2………………………………………………………………………………………………………….7

# Supplemental Table I. First pass vs. Final TICI results

|  | **Final TICI 0-1** | **Final TICI 2a** | **Final TICI 2b** | **Final TICI 2c** | **Final TICI 3** |
| --- | --- | --- | --- | --- | --- |
| FP TICI 0-1 (n=53) | 6  (11.3%) | 1  (1.9%) | 8  (15.1%) | 15  (28.3%) | 23  (43.4%) |
| FP TICI 2a (n=27) | 1  (3.7%) | 4  (14.8%) | 7  (25.9%) | 6  (22.2%) | 9  (33.3%) |
| FP TICI 2b (n=19) | 0 | 0 | 13  (68.4%) | 3  (15.8%) | 3  (15.8%) |
| FP TICI 2c (n=18) | 0 | 0 | 0 | 18  (100%) | 0 |
| FP TICI 3 (n=44) | 0 | 0 | 0 | 0 | 44  (100%) |

Abbreviation: FP, first-pass; TICI, thrombolysis in cerebral infarction.

# Supplemental Table II. Association of baseline variables with clinical and final angiographic outcomes

| **Variable** | **90-day**  **mRS 0-2**  **(n=103)** | **90-day**  **mRS 3-6**  **(n=53)** | **P-value** | **Final TICI**  **2c-3**  **(n=121)** | **Final**  **TICI 0-2b (n=40)** | **P-value** |
| --- | --- | --- | --- | --- | --- | --- |
| **Age (years); mean ± SD** | 65.1 ± 14.0 | 70.2 ± 11.5 | 0.02 | 66.9 ± 13.3 | 66.2 ± 13.3 | 0.78 |
| **Female sex; n (%)** | 61 (59.2%) | 29 (54.7%) | 0.59 | 72 (59.5%) | 20 (50%) | 0.29 |
| **Baseline NIHSS score; median (IQR)** | 15 (12-18) | 19 (16-22) | <0.0001 | 17 (13-20) | 15 (12-19.5) | 0.32 |
| **Baseline NCCT ASPECTS; median (IQR)** | 10 (10-10)  (n=83) | 10 (9-10)  (n=37) | 0.03 | 10 (9-10)  (n=94) | 10 (10-10)  (n=30) | 0.47 |
| **Occlusion level; n (%)**  **ICA**  **MCA M1** | 18 (17.5%)  85 (82.5%) | 16 (30.2%)  37 (69.8%) | 0.07 | 27 (22.3%)  94 (77.7%) | 8 (20.0%)  32 (80.0%) | 0.76 |
| **IV tPA treatment; n (%)** | 69 (67.0%) | 33 (62.3%) | 0.56 | 82 (67.8%) | 24 (60.0%) | 0.37 |
| **Hypertension; n (%)** | 70 (68.0%) | 34 (64.2%) | 0.63 | 84 (69.4%) | 23 (57.5%) | 0.17 |
| **Diabetes mellitus; n (%)** | 17 (16.5%) | 14 (26.4%) | 0.14 | 23 (19.0%) | 8 (20.0%) | 0.89 |
| **Atrial fibrillation; n (%)** | 33 (32.0%) | 27 (50.9%) | 0.02 | 47 (38.8%) | 15 (37.5%) | 0.88 |
| **Dyslipidemia; n (%)** | 39 (37.9%) | 22 (41.5%) | 0.66 | 48 (39.7%) | 15 (37.5%) | 0.81 |
| **Smoking; n (%)** | 26 (25.2%) | 12 (22.6%) | 0.72 | 31 (25.6%) | 11 (27.5%) | 0.82 |
| **Previous stroke/transient ischemic attack; n (%)** | 15 (14.6%) | 12 (22.6%) | 0.21 | 20 (16.5%) | 9 (22.5%) | 0.40 |
| **Previous MI/CAD; n (%)** | 24 (23.3%) | 10 (18.9%) | 0.53 | 29 (24.0%) | 6 (15.0%) | 0.23 |

Abbreviations: mRS, modified Rankin Scale; TICI, Treatment in Cerebral Ischemia scale; SD, standard deviation; NIHSS, National Institutes of Health Stroke Scale; NCCT ASPECTS, non-contrast CT Alberta Stroke Program Early CT Score; IQR, interquartile range; MI/CAD, myocardial infarction/coronary artery disease.

# Supplemental Table III. Number of passes to final TICI 2c-3 vs. 90-day functional outcome in early- vs. late-treated patients.

| **Variable** | **1 pass** | **2 passes** | **3 passes** | **4+ passes** | **P-value** |
| --- | --- | --- | --- | --- | --- |
| **≤4 hours to groin puncture from stroke onset (n=83)** |  |  |  |  |  |
| 90-day mRS; median (IQR) | 0 (0-2)  (n=45) | 1 (1-2)  (n=13) | 1.5 (0.5-5.5)  (n=8) | 2 (1-4)  (n=15) | 0.0003* |
| 90-day mRS 0-2; n (%) | 37/45 (82%) | 10/13 (77%) | 5/8 (63%) | 9/15 (60%) | 0.06° |
| **>4 hours to groin puncture from stroke onset (n=38)** |  |  |  |  |  |
| 90-day mRS; median (IQR) | 1.5 (1-2)  (n=14) | 2.5 (0.5-4.5)  (n=8) | 2.5 (2-3)  (n=10) | 4.5 (1.5-6)  (n=4) | 0.09* |
| 90-day mRS 0-2; n (%) | 11/14 (79%) | 4/8 (50%) | 5/10 (50%) | 1/4 (25%) | 0.04° |

Abbreviations: TICI, Treatment in Cerebral Ischemia scale; mRS, modified Rankin Scale; IQR, interquartile range. *Jonckheere-Terpstra trend test. °Chi-squared trend test

# Supplemental Figure I: Study Flowchart

4,792 ARISE II patients screened

Excluded (n=4,565)

  Not meeting inclusion criteria (n=4,548)

  Not meeting angiographic eligibility (n=16)

  Other reasons (n=1)

161 patients analyzed in this study

Excluded (n=66)

  Patients with basilar occlusion (n=9)

  Patients with M2 occlusion (n=57)

227 patients treated in ARISE II (n227) )

# Supplemental Figure II.

ROC Curve for Final TICI (0-2a vs. 2b vs. 2c vs. 3) versus 90-day mRS 0-2

| **Criterion** | Sensitivity | 95% CI | Specificity | 95% CI |
| --- | --- | --- | --- | --- |
| 0-3 | 100 | 96.5-100 | 0 | 0-6.7 |
| 2b-3 | 95.2 | 89.0-98.4 | 13.2 | 5.5-25.3 |
| **2c-3** | **79.6** | **70.5-86.9** | **34.0** | **21.5-48.3** |
| 3 | 51.5 | 41.4-61.4 | 54.7 | 40.4-68.4 |

**Supplemental Figure III.**

ROC Curve for Number of Passes to TICI 2c-3 (1 vs. ≤2 vs. ≤3 vs. 4+) versus 90-day mRS 0-2

| **Criterion** | Sensitivity | 95% CI | Specificity | 95% CI |
| --- | --- | --- | --- | --- |
| **1** | **58.5** | **47.1-69.3** | **68.6** | **50.7-83.1** |
| ≤2 | 75.6 | 64.9-84.4 | 48.6 | 31.4-66.0 |
| ≤3 | 87.8 | 78.7-94.0 | 25.7 | 12.5-43.3 |
| 4+ | 100 | 95.6-100 | 0 | 0-10.0 |
