# Supplementary material for: Benchmarking the Extent and Speed of Reperfusion: First Pass TICI 2c-3 Is a Preferred Endovascular Reperfusion Endpoint
Source: Front Neurol. 2021 May 11;12:669934. doi: 10.3389/fneur.2021.669934 (PMC8144635; doi:10.3389/fneur.2021.669934)
Supplement: Supplementary file 2 [file Data_Sheet_2.pdf]

ARISE II Investigators

First	M.I.	Last	Highest degree	Institution	City, Country	Function
René		Chapot	MD	Alfried Krupp Krankenhaus	Essen, Germany	Steering Committee, Site PI
Ana	P	Narata	MD	CHRU Hôpitaux de Tours	Tours, France	Steering Committee, Site PI
Jonathan	A	Grossberg	MD	Emory University School of Medicine	Atlanta, GA, USA	Steering Committee, Site PI
Raul	G	Nogueira	MD	Emory University School of Medicine	Atlanta, GA, USA	Steering Committee, Site Investigator
Tudor	G	Jovin	MD	University of Pittsburgh Medical Center	Pittsburgh, PA, USA	Steering Committee, Site Investigator
Adnan	H	Siddiqui	MD, PhD	University of Buffalo	New York, NY, USA	Steering Committee, Site PI
Mairsil		Claffey	-	Cerenovus	Galway, Ireland	Steering Committee
Steven	W	Hetts	MD	University of California	San Fransisco, CA	Data Safety Monitoring Board, Clinical Events Committee
Werner		Hacke	MD, PhD	University of Heidelberg	Heidelberg, Germany	Data Safety Monitoring Board
Brijesh	P	Mehta	MD	South Broward Hospital	Hollywood, FL	Data Safety Monitoring Board
Lofti		Hacein-Bey	MD	University of California Davis School of Medicine	Sacramento, CA	Data Safety Monitoring Board, Clinical Events Committee
Anthony	W	Kim	MD	Keck School of Medicine, University of Southern California	Los Angeles, CA	Data Safety Monitoring Board, Clinical Events Committee
Alex		Abou-Chebl	MD	Baptist Health System	Louisville, KY	Data Safety Monitoring Board, Clinical Events Committee
Peter		Shabe	MSc	Advance Research Associates, Inc	Santa Clara, CA	Data Safety Monitoring Board, Biostatistician
Ryan	A	Priest	MD	Oregon Health and Science University Hospital	Portland, OR, USA	Site Investigator

Gary	M	Nesbit	MD	Oregon Health and Science University Hospital	Portland, OR, USA	Site Investigator
Wayne	M	Clark	MD	Oregon Health and Science University Hospital	Portland, OR, USA	Site Investigator
Masahiro		Horikawa	MD	Oregon Health and Science University Hospital	Portland, OR, USA	Site Investigator
David	A	Hoak	MD	Oregon Health and Science University Hospital	Portland, OR, USA	Site Investigator
Bryan	D	Petersen	MD	Oregon Health and Science University Hospital	Portland, OR, USA	Site Investigator
Noah	C	Beadell	MD	Oregon Health and Science University Hospital	Portland, OR, USA	Site Investigator
Kory	S	Herrick	MD	Oregon Health and Science University Hospital	Portland, OR, USA	Site Investigator
Corey	R	White	DO	Oregon Health and Science University Hospital	Portland, OR, USA	Site Investigator
Michelle	T	Stacey	MD	Oregon Health and Science University Hospital	Portland, OR, USA	Site Investigator
Sierra	C	Ford	DO	Oregon Health and Science University Hospital	Portland, OR, USA	Site Investigator
Jesse	J	Liu	MD	Oregon Health and Science University Hospital	Portland, OR, USA	Site Investigator
Alejandro		Tomasello	MD	Hospital Vall d'Hebron	Barcelona, Spain	Site Investigator
Carlos	A	Molina	MD, PhD	Hospital Vall d'Hebron	Barcelona, Spain	Site Investigator
David		Rodriguez-Luna	MD, PhD	Hospital Vall d'Hebron	Barcelona, Spain	Site Investigator
Sandra		Boned-Riera	MD	Hospital Vall d'Hebron	Barcelona, Spain	Site Investigator
Jorge		Pagola	MD, PhD	Hospital Vall d'Hebron	Barcelona, Spain	Site Investigator
Marta		Rubiera	MD, PhD	Hospital Vall d'Hebron	Barcelona, Spain	Site Investigator
Jesus	M	Juega	MD	Hospital Vall d'Hebron	Barcelona, Spain	Site Investigator
Noelia		Rodriguez-Villatoro	MD	Hospital Vall d'Hebron	Barcelona, Spain	Site Investigator
Hannes		Nordmeyer	MD	Alfried Krupp Krankenhaus	Essen, Germany	Site Investigator
Michael		Stauder	-	Alfried Krupp Krankenhaus	Essen, Germany	Site Investigator
Christian	P	Stracke	MD	Alfried Krupp Krankenhaus	Essen, Germany	Site Investigator

Markus		Heddier	-	Alfried Krupp Krankenhaus	Essen, Germany	Site Investigator
Denis		Herbreteau	MD, PhD	CHRU Hôpitaux de Tours	Tours, France	Site Investigator
Richard		Bibi	MD	CHRU Hôpitaux de Tours	Tours, France	Site Investigator
Oliver		Francois	MD	AZ Groeninge	Kortrijk, Belgium	Site Investigator
D		Pieters	-	AZ Groeninge	Kortrijk, Belgium	Site Investigator
Tom		Dewaele	MD	AZ Groeninge	Kortrijk, Belgium	Site Investigator
Paul		Bourgeois	-	AZ Groeninge	Kortrijk, Belgium	Site Investigator
Frederik		Vanhee	-	AZ Groeninge	Kortrijk, Belgium	Site Investigator
P atrick		Vanderdouckt	-	AZ Groeninge	Kortrijk, Belgium	Site Investigator
Evelien		Vancaester	-	AZ Groeninge	Kortrijk, Belgium	Site Investigator
Tudor	G	Jovin	MD	University of Pittsburgh Medical Center	Pittsburgh, PA, USA	Site Investigator
Brian	T	Jankowitz	MD	University of Pittsburgh Medical Center	Pittsburgh, PA, USA	Site Investigator
Andrew	F	Ducruet	MD	University of Pittsburgh Medical Center	Pittsburgh, PA, USA	Site Investigator
Amin	N	Aghaebrahim	MD	University of Pittsburgh Medical Center	Pittsburgh, PA, USA	Site Investigator
Cynthia	L	Kenmuir	MD, PhD	University of Pittsburgh Medical Center	Pittsburgh, PA, USA	Site Investigator
Hazem	M	Shoirah	MD	University of Pittsburgh Medical Center	Pittsburgh, PA, USA	Site Investigator
Bradley	J	Molyneaux	MD, PhD	University of Pittsburgh Medical Center	Pittsburgh, PA, USA	Site Investigator
Prasanna	K	Tadi	MD	University of Pittsburgh Medical Center	Pittsburgh, PA, USA	Site Investigator
Gena	M	Walker	MD	University of Pittsburgh Medical Center	Pittsburgh, PA, USA	Site Investigator
Matthew	T	Starr	MD	University of Pittsburgh Medical Center	Pittsburgh, PA, USA	Site Investigator
Diogo	C	Haussen	MD	Emory University School of Medicine	Atlanta, GA, USA	Site Investigator
Michael	R	Frankel	MD	Emory University School of Medicine	Atlanta, GA, USA	Site Investigator

Nicolas	A	Bianchi	MD	Emory University School of Medicine	Atlanta, GA, USA	Site Investigator
Samir	R	Belegaje	MD	Emory University School of Medicine	Atlanta, GA, USA	Site Investigator
Nicole	D	Mahdi	MD	Emory University School of Medicine	Atlanta, GA, USA	Site Investigator
Sourabh		Lahoti	MD	Emory University School of Medicine	Atlanta, GA, USA	Site Investigator
Anna	N	Katema	DO	Emory University School of Medicine	Atlanta, GA, USA	Site Investigator
Melanie	J	Winngingham	MD	Emory University School of Medicine	Atlanta, GA, USA	Site Investigator
Aaron	M	Anderson	MD	Emory University School of Medicine	Atlanta, GA, USA	Site Investigator
Eugene		Lin	MD	Mercy St. Vincent Medical Center	Toldo, OH, USA	Site Investigator
Christian	H	Riedel	MD	Universitätsklinikum Schleswig-Holstein, Kiel Campus	Kiel, Germany	Site PI
Olav		Jansen	MD, PhD	Universitätsklinikum Schleswig-Holstein, Kiel Campus	Kiel, Germany	Site Investigator
Fritz		Wodarg	MD	Universitätsklinikum Schleswig-Holstein, Kiel Campus	Kiel, Germany	Site Investigator
Naomi		Larsen	MD	Universitätsklinikum Schleswig-Holstein, Kiel Campus	Kiel, Germany	Site Investigator
Andreas		Binder	MD	Universitätsklinikum Schleswig-Holstein, Kiel Campus	Kiel, Germany	Site Investigator
Daniel		Wiesen	MSc	Universitätsklinikum Schleswig-Holstein, Kiel Campus	Kiel, Germany	Site Investigator
Kenneth	V	Snyder	MD, PhD	University of Buffalo	New York, NY, USA	Site Investigator
Elad	I	Levy	MD	University of Buffalo	New York, NY, USA	Site Investigator
Jason	M	Davies	MD, PhD	University of Buffalo	New York, NY, USA	Site Investigator

Ashish		Sonig	MD	University of Buffalo	New York, NY, USA	Site Investigator
Leonardo	N	Rangel-Castilla	MD	University of Buffalo	New York, NY, USA	Site Investigator
Ashkan		Mowla	MD	University of Buffalo	New York, NY, USA	Site Investigator
Hakeem	J	Shakir	MD	University of Buffalo	New York, NY, USA	Site Investigator
Vernard	S	Fennel	MD, MSc	University of Buffalo	New York, NY, USA	Site Investigator
Gursant	S	Atwal	MD	University of Buffalo	New York, NY, USA	Site Investigator
Sabareesh	K	Natarajan	MD, MSc	University of Buffalo	New York, NY, USA	Site Investigator
J		Beecher	-	University of Buffalo	New York, NY, USA	Site Investigator
John		Thornton	MB FFR RCSI	Beaumont Hospital	Dubline, Ireland	Site PI
Paul		Brennan	MRCPI FRCR FFRRCSI MSc	Beaumont Hospital	Dubline, Ireland	Site Investigator
Alan		O'Hare	MB BCh BAO	Beaumont Hospital	Dubline, Ireland	Site Investigator
Hamed		Asadi	MD, PhD	Beaumont Hospital	Dubline, Ireland	Site Investigator
Ronald	F	Budzik	MD	Riverside Radiology and Interventional Associates	Columbus, USA	Site PI
N		Voraco	-	Riverside Radiology and Interventional Associates	Columbus, USA	Site Investigator
Peter	J	Pema	MD	Riverside Radiology and Interventional Associates	Columbus, USA	Site Investigator
Thomas	M	Davis	MD	Riverside Radiology and Interventional Associates	Columbus, USA	Site Investigator
William	J	Hicks	MD	Riverside Radiology and Interventional Associates	Columbus, USA	Site Investigator
Jennifer	D	Mejilla	DO	Riverside Radiology and Interventional Associates	Columbus, USA	Site Investigator
Mohamed	S	Teleb	MD	Banner Desert Medical Center	AZ, USA	Site PI
Peter	J	Sunenshine	MD	Banner Desert Medical Center	AZ, USA	Site PI
Jacqueline	M	Carter	MD	Banner Desert Medical Center	AZ, USA	Site Investigator

Christian	A	Taschner	MD	Universitätsklinikum Freiburg	Freiburg, Germany	Site PI
Stephan		Meckel	MD	Universitätsklinikum Freiburg	Freiburg, Germany	Site Investigator
Samer		Elsheik	MD	Universitätsklinikum Freiburg	Freiburg, Germany	Site Investigator
Horst		Urbach	MD	Universitätsklinikum Freiburg	Freiburg, Germany	Site Investigator
Christoph	A	Maurer	MD, FACS, FRCS, FEBS	Universitätsklinikum Freiburg	Freiburg, Germany	Site Investigator
Karl		Egger	MD	Universitätsklinikum Freiburg	Freiburg, Germany	Site Investigator
Wolf	D	Nieson	MD	Universitätsklinikum Freiburg	Freiburg, Germany	Site Investigator
Blaise	W	Baxter	MD	Erlanger Hospital	Chattanooga, TN	Site PI
Steven	D	Quarfordt	MD	Erlanger Hospital	Chattanooga, TN	Site Investigator
Justin	A	Calvert	MD	Erlanger Hospital	Chattanooga, TN	Site Investigator
Harris	E	Hawk	MD	Erlanger Hospital	Chattanooga, TN	Site Investigator
Reza		Malek	MD, Msc	Good Samaritan Hospital / Regional Medical Center	San Jose, CA	Site PI
Arash	M	Padidar	MD	Good Samaritan Hospital / Regional Medical Center	San Jose, CA	Site PI
Ursula		Kelly-Tolley	RN	Good Samaritan Hospital / Regional Medical Center	San Jose, CA	Site Investigator
A		Gutierrez	-	Good Samaritan Hospital / Regional Medical Center	San Jose, CA	Site Investigator
Pasquale		Mordasini	MD, Msc	Inselspital, University Hospital Bern	Bern, Switzerland	Site PI
Rupashani		Balasubramaniam	-	Inselspital, University Hospital Bern	Bern, Switzerland	Site Investigator
Jan		Gralla	MD	Inselspital, University Hospital Bern	Bern, Switzerland	Site Investigator

Urs		Fischer	MD	Inselspital, University Hospital Bern	Bern, Switzerland	Site Investigator
Felix		Zibold	MD	Inselspital, University Hospital Bern	Bern, Switzerland	Site Investigator
Eike	I	Piechowiak	MD	Inselspital, University Hospital Bern	Bern, Switzerland	Site Investigator
Reade	A	DeLeacy	MD	Ichan School of Medicine at Mount Sinai	New York, USA	Site PI
Johanna	T	Fifi	MD	Ichan School of Medicine at Mount Sinai	New York, USA	Site Investigator
Jay		Mocco	MD, Msc	Ichan School of Medicine at Mount Sinai	New York, USA	Site Investigator
Sidney		Starkman	MD	UCLA Stroke Network	Los Angeles, CA	Site PI
Viktor		Szeder	MD, PhD, MSc	UCLA Stroke Network	Los Angeles, CA	Site Investigator
Satoshi		Tateshima	MD	UCLA Stroke Network	Los Angeles, CA	Site Investigator
Gary	R	Duckwiler	MD	UCLA Stroke Network	Los Angeles, CA	Site Investigator
May		Nour	MD, PhD	UCLA Stroke Network	Los Angeles, CA	Site Investigator
Xian	N	Tang	MD	UCLA Stroke Network	Los Angeles, CA	Site Investigator
Jason	D	Hinman	MD, PhD	UCLA Stroke Network	Los Angeles, CA	Site Investigator
Anita		Tipirneni	MD	UCLA Stroke Network	Los Angeles, CA	Site Investigator
Dileep	R	Yavagal	MD	University of Miami / Jackson Memorial	Miami, FL, USA	Site PI
S		Suir	-	University of Miami / Jackson Memorial	Miami, FL, USA	Site Investigator
Justin	M	Caplan	MD	University of Miami / Jackson Memorial	Miami, FL, USA	Site Investigator
P		Kandewall	-	University of Miami / Jackson Memorial	Miami, FL, USA	Site Investigator
Eric	C	Peterson	MD	University of Miami / Jackson Memorial	Miami, FL, USA	Site Investigator
Robert	M	Starke	MD	University of Miami / Jackson Memorial	Miami, FL, USA	Site Investigator
Ajit	S	Puri	MD	UMASS Memorial Medical	Worchester, MA, USA	Site PI

David	E	Rex	MD, PhD	UMASS Memorial Medical	Worchester, MA, USA	Site Investigator
Francesco		Massari	MD, PhD	UMASS Memorial Medical	Worchester, MA, USA	Site Investigator
Ajay	K	Wakhloo	MD, PhD	UMASS Memorial Medical	Worchester, MA, USA	Site Investigator
Juan	D	Lozano	MD	UMASS Memorial Medical	Worchester, MA, USA	Site Investigator
Katyucia	D	Rodrigues	MD	UMASS Memorial Medical	Worchester, MA, USA	Site Investigator
Laurent		Pierot	MD, PhD	CHRU de Reims, Hôpital Maison Blanche	Reims, France	Site PI
		Bouquigyn	-	CHRU de Reims, Hôpital Maison Blanche	Reims, France	Site Investigator
S		Sebastien	-	CHRU de Reims, Hôpital Maison Blanche	Reims, France	Site Investigator
M	G	Emmoinoli	-	CHRU de Reims, Hôpital Maison Blanche	Reims, France	Site Investigator
